# Acute Pancreatitis Associated With Systemic Lupus Erythematosus in a Young Female: A Diagnostic and Therapeutic Challenge

**DOI:** 10.1002/ccr3.9621

**Published:** 2024-11-23

**Authors:** Andres Ordoñes‐Saucedo, Bruno Eduardo Reyes‐Torres, Karen Kortright‐Maldonado, Erika K. Tenorio‐Aguirre, Pedro Rodríguez‐Henríquez, Froylan D. Martínez‐Sánchez

**Affiliations:** ^1^ Department of Internal Medicine Hospital General Dr. Manuel Gea Gonzalez Ciudad de México Mexico; ^2^ Facultad de Medicina Universidad Nacional Autónoma de México Ciudad de México Mexico; ^3^ Department of Rheumatology Hospital General Dr. Manuel Gea Gonzalez Ciudad de México Mexico

**Keywords:** abdominal pain, acute pancreatitis, gastrointestinal, systemic lupus erythematosus

## Abstract

Acute pancreatitis (AP) is a rare but life‐threatening complication in patients with systemic lupus erythematosus (SLE). The case highlights the diagnostic challenges and treatment complexities in managing SLE‐associated pancreatitis. A 20‐year‐old female with a history of SLE presented with acute onset epigastric pain, vomiting, and signs of systemic inflammation. Laboratory findings revealed elevated amylase and lipase levels, confirming AP. Imaging studies showed interstitial edematous pancreatitis and bilateral pleural effusion. The patient was managed with aggressive fluid resuscitation, pain management, and supportive care. A systemic inflammatory response complicated her clinical course, and she required intensive care unit monitoring. This case underscores the importance of early recognition of AP in SLE patients and highlights the need for a multidisciplinary approach to manage this severe complication.

## Graphical abstract

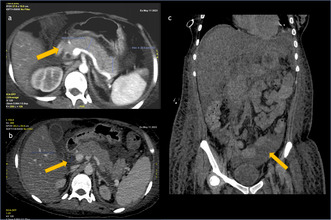

**Summary**

- Acute pancreatitis is a rare but severe complication of systemic lupus erythematosus.
- Early recognition and a multidisciplinary approach are critical in managing this life‐threatening condition to improve patient outcomes.

## 1 Introduction

Systemic lupus erythematosus (SLE) is a complex, multisystem autoimmune disorder that affects various organs, including the skin, joints, kidneys, and nervous system [1, 2]. Among the many manifestations of SLE, gastrointestinal involvement is relatively common, ranging from mild symptoms such as serositis and elevated liver enzymes to life‐threatening conditions like mesenteric vasculitis and acute pancreatitis (AP) [2, 3]. AP is a rare but severe complication of SLE, with a reported incidence ranging from 0.6% to 3.5% of patients with the disease [2, 4]. Although uncommon, AP can be life‐threatening, mainly when it occurs in the context of active SLE [2, 4].

The pathogenesis of AP in SLE has yet to be fully understood, but several mechanisms have been proposed [3, 5]. These include small vessel vasculitis, immune complex deposition, and microthrombi formation, which can lead to ischemia of the pancreas. In some cases, the development of AP in SLE may also be associated with the use of medications, such as corticosteroids and immunosuppressive drugs like azathioprine [3]. However, many episodes are classified as idiopathic, with no apparent underlying cause other than active SLE [2].

Patients with SLE who develop AP often present with severe abdominal pain, vomiting, and signs of systemic inflammation [3, 4]. Studies have shown that AP in the setting of SLE tends to occur early in the course of the disease and is frequently associated with lupus nephritis, neuropsychiatric lupus, and other systemic complications [3, 4, 5]. The mortality rate for SLE‐associated AP remains high, with severe cases often complicated by multiorgan failure and sepsis [4, 5]. This case report highlights the challenges of diagnosing and managing AP in a young woman with SLE, illustrating the importance of early recognition and aggressive treatment to improve patient outcomes.

## 2 Case History/Examination

A 20‐year‐old woman from Chimalhuacan, Mexico, presented with a history of SLE, diagnosed recently during her hospital stay. Her past medical history includes major depressive disorder, treated with sertraline and pregabalin, and a previous episode of left hemiparesis in July 2021, from which she had fully recovered. Her family history was notable for her father's death due to chronic kidney disease and her grandmother's diabetes and hypertension.

Her current condition began on April 15, 2023, with symptoms of asthenia and adynamia that progressed to weakness in her lower extremities, eventually leading to immobility. Over the following weeks, she developed significant lower limb edema, for which she sought medical attention on April 22, 2023. She was prescribed furosemide with partial improvement but did not undergo further investigations. By May 4, 2023, she experienced severe, colicky epigastric pain rated 10/10, accompanied by nausea, vomiting, dysuria, and urinary urgency. She sought medical attention the following day at our institution and was admitted to the emergency department.

On presentation, her vital signs were stable, with a blood pressure of 131/71 mmHg, a heart rate of 90 beats per minute, a respiratory rate of 22 breaths per minute, oxygen saturation of 93%, and a temperature of 36.5°C. She was somnolent and disoriented, with a Glasgow Coma Scale score of 13.

## 3 Methods (Differential Diagnosis, Investigations, and Treatment)

### 3.1 Differential Diagnosis

The primary differential diagnosis was AP due to the patient's severe abdominal pain and vomiting, with possible gastrointestinal complications related to her SLE. Other differentials considered included urinary tract infection due to dysuria and urinary urgency and potential lupus nephritis complications, given her history of SLE.

### 3.2 Investigations

**FIGURE 1:**
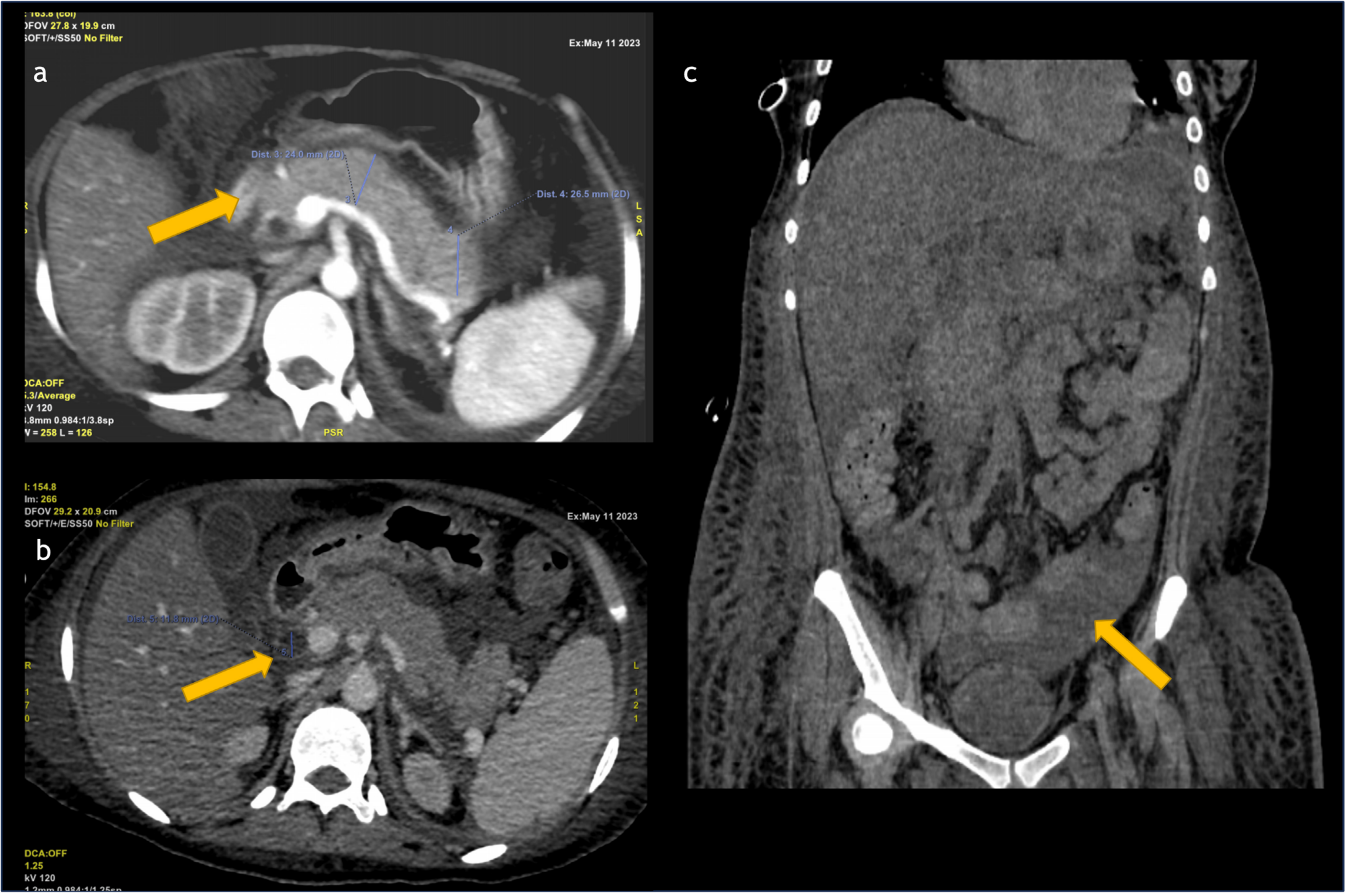
(a) Contrast‐enhanced computed tomography (CT) image of the pancreas showing significant pancreatic enlargement with a hypodense area measuring 24.0 mm (yellow arrow), suggestive of acute inflammation or necrosis. There is notable peripancreatic fat stranding and fluid collection, indicating acute pancreatitis with a potential developing pseudocyst. (b) Axial CT image of the abdomen demonstrating an enlarged pancreas with an inflamed area measuring 11.8 mm (yellow arrow). The image shows peripancreatic fluid accumulation and fat stranding, consistent with acute interstitial edematous pancreatitis. (c) Coronal CT image showing free fluid in the abdominal cavity (yellow arrow), commonly associated with acute pancreatitis.

Laboratory results revealed an elevated lipase level of 2917 U/L and amylase of 352 U/L, confirming a diagnosis of AP. Imaging studies, including a contrast‐enhanced CT scan of the abdomen, revealed interstitial edematous pancreatitis, moderate pericardial and pleural effusions, and free fluid in the abdomen (Figure 1). Further investigations confirmed SLE with renal involvement (class IV/V lupus nephritis), serological positivity for anti‐double‐stranded DNA, and anti‐Smith antibodies. She was also diagnosed with antiphospholipid syndrome based on positive lupus anticoagulant tests and elevated anticardiolipin IgM antibodies (Table 1). The Systemic Lupus Erythematosus Disease Activity Index (SLEDAI) score was 38 points, indicating severe disease activity.

**TABLE 1:** Autoantibodies profile.

Study	Result	Units	Reference range
Antinuclear Antibodies Hep‐2 cell substrate	1:1280 Nuclear homogeneous dilution		Positive > 1:40
Anti‐SSA (Ro 52)	< 2.3	CU	Adult: 2.3–20 CU
Anti‐SSA (Ro 60)	15.3	CU	Adult: 4.9–20 CU
Anti‐RNP	3.6	CU	Adult: 3.5–20 CU
Anti‐Sm (Smith)	3.8	CU	Adult: 3.3–20 CU
Anti‐SSB	< 3.3	CU	Adult: 3.3–20 CU
Anti‐DNA (double‐stranded)	2511.9	UI/ml	Adult: 9.8–27 UI/mL

### 3.3 Treatment

The patient received pulse‐dose corticosteroids (methylprednisolone 500 mg/day in three doses), intravenous cyclophosphamide (calculated dose of 0.75 g/m^2^), and hydroxychloroquine 200 mg/day to control her lupus flare. Her AP was managed conservatively with bowel rest, intravenous fluids, and pain control.

Her hospital course was complicated by acute kidney injury, severe hyperkalemia, and metabolic acidosis, necessitating fluid resuscitation, vasopressor support, and continuous renal replacement therapy. Hemodialysis was initiated due to worsening renal function, with creatinine levels peaking at 5.7 mg/dL. Throughout her hospitalization, she developed multiple complications, including recurrent infections, requiring broad‐spectrum antibiotics and antifungal therapy. On June 23, 2023, she underwent placement of a Tenckhoff catheter for peritoneal dialysis, as her renal function did not recover sufficiently to discontinue dialysis.

## 4 Conclusion and Results (Outcome and Follow‐Up)

Despite these complications, the patient gradually improved with aggressive supportive care. Over time, her renal function stabilized, and her pleural and pericardial effusions resolved. She remained dependent on intermittent hemodialysis for persistent renal failure. She was discharged on July 14, 2023, with a multidisciplinary follow‐up plan that included nephrology, rheumatology, and internal medicine.

At discharge, she was prescribed prednisone, hydroxychloroquine, and anticoagulation therapy for her antiphospholipid syndrome. Her condition at discharge was significantly improved, although she remained on peritoneal dialysis and continued treatment for her underlying SLE.

This case illustrates the complex management required for patients with SLE who present with multi‐organ involvement, including AP, lupus nephritis, and antiphospholipid syndrome. Early recognition and a multidisciplinary approach are crucial in preventing further morbidity and improving outcomes in these patients.

## 5 Discussion

AP in patients with SLE is a rare but serious complication [3, 4, 5]. Pascual‐Ramos et al. reported a 3.5% prevalence of AP in a large cohort of SLE patients, with idiopathic cases constituting a significant portion of these episodes, particularly in those with higher disease activity (as reflected by Mex‐SLEDAI scores) [2]. Moreover, the clinical course of SLE is marked by systemic involvement and unpredictable flares, requiring a high index of suspicion for organ‐specific manifestations such as AP [1, 3]. The pathogenesis of AP in SLE remains complex, with mechanisms involving vascular damage and immune‐mediated inflammation, making timely diagnosis and management essential for improving outcomes in these patients [2, 3, 4, 5].

Pancreatitis in SLE is often associated with vasculitis, immune complex deposition, and thrombosis, all of which contribute to tissue necrosis and inflammation in the pancreas [8]. AP is a life‐threatening condition that demands early recognition to carry out aggressive fluid management. Tools such as BISAP and APACHE II can be utilized for severity stratification of AP [6, 7]. In patients with SLE, AP can be particularly challenging due to the underlying autoimmune activity, which complicates the clinical course and increases the risk of multi‐organ involvement [2, 6, 7, 8].

The present case is consistent with reports in the literature. Similar to other reported cases, abdominal pain and elevated pancreatic enzymes were the hallmark signs of AP, and the diagnosis was supported by imaging findings [7, 8]. Muhammed et al. reported that AP in SLE is typically associated with active disease, especially within the first year of diagnosis, as in our case, where the patient had significant disease activity at the time of AP onset [4]. Our patient's age, gender, and the presence of nephritis mirror findings from large cohort studies, which also indicate that young women with SLE, particularly those with nephritis, are at a higher risk of developing AP [4]. Studies by Muhammed et al. and others report that AP in SLE frequently occurs in the setting of active nephritis and low complement levels (C3 and C4), both of which were evident in our patients [3, 4, 9, 10]. The literature suggests that lupus‐related mechanisms, such as vasculitis, immune complex deposition, and ischemia, contribute to the pathogenesis of AP, which may explain the complexity and multi‐system involvement observed in our patient [4].

Moreover, Alina Dima et al. expanded upon the mechanisms underlying AP in lupus, describing it as a result of immune‐mediated damage, including vasculitis, ischemia, and immune complex deposition in pancreatic tissue [3]. This autoimmune involvement is a key factor in the development of AP, distinguishing it from other causes of pancreatitis [2, 3]. In our case, the patient's elevated anti‐dsDNA, low complement levels (C3 and C4), and the presence of lupus nephritis align with these pathophysiological mechanisms [3, 4].

The association between AP and macrophage activation syndrome (MAS) was noted by Lin et al., who reported a high prevalence of MAS in pediatric lupus patients with AP [5]. Although our patient did not develop MAS, the systemic nature of her disease, characterized by renal failure and immune system activation, resembles the high morbidity reported in such cases [5]. The need for aggressive interventions, including renal replacement therapy and broad‐spectrum antibiotics for recurrent infections, reflects the serious nature of AP in SLE patients, as documented by Dima et al. and others [3, 5].

In terms of outcomes, our patient's long hospital stay, recurrent infections, and dependence on dialysis mirror the prolonged and complex recoveries observed in similar cases [8]. Dima et al. emphasized that while survival is possible with early and aggressive treatment, long‐term complications such as renal failure and the need for dialysis can persist, as seen in our patient [3]. Additionally, it has been reported early mortality rates are as high as 17%, reinforcing the critical nature of timely intervention [4].

This case highlights several critical features of SLE‐associated AP [2, 8]. First, the disease was managed in the setting of active lupus, with high SLEDAI scores, supporting the idea that SLE‐related immune mechanisms likely contributed to AP. Second, the patient required a multidisciplinary approach, including rheumatology and nephrology consultation, as her case involved multiple organs. The immunosuppressive treatment was tailored based on clinical severity, following the current clinical practice guidelines for SLE management.

The primary limitation in this case was the inability to perform a biopsy or direct histopathological evaluation of the pancreas to confirm the presence of vasculitis, a known cause of SLE‐associated AP [8]. Additionally, while the patient was treated with corticosteroids and cyclophosphamide, well‐documented therapies for controlling SLE flares, there remains a lack of randomized controlled trials to standardize the treatment protocols specifically for SLE‐associated AP [4]. Lastly, the lack of long‐term follow‐up data on pancreatic function in this patient limits the understanding of potential chronic pancreatic damage or the recurrence of AP.

## 6 Conclusion

This case emphasizes the complexity of managing AP in patients with SLE. Early recognition, prompt intervention, and a multidisciplinary approach are critical to improving outcomes in these patients.

## Author Contributions

**Andres Ordoñes‐Saucedo:** conceptualization, formal analysis, investigation, visualization, writing – original draft, writing – review and editing. **Bruno Eduardo Reyes‐Torres:** investigation, methodology, project administration, writing – original draft, writing – review and editing. **Karen Kortright‐Maldonado:** investigation, methodology, validation, writing – original draft, writing – review and editing. **Erika K. Tenorio‐Aguirre:** funding acquisition, project administration, writing – original draft, writing – review and editing. **Pedro Rodríguez‐Henríquez:** conceptualization, data curation, supervision, validation, visualization, writing – original draft, writing – review and editing. **Froylan D. Martínez‐Sánchez:** conceptualization, formal analysis, funding acquisition, methodology, project administration, supervision, validation, visualization, writing – original draft, writing – review and editing.

## Disclosure

The authors have nothing to report.

## Consent

Signed informed consent was obtained directly from the patient.

## Conflicts of Interest

The authors declare no conflicts of interest.

## Data Availability

The data that support the findings of this study are available from the corresponding author upon reasonable request.
