# Correction to “Acute Pancreatitis Associated With Systemic Lupus Erythematosus in a Young Female: A Diagnostic and Therapeutic Challenge”

**DOI:** 10.1002/ccr3.9702

**Published:** 2024-12-05

A. Ordoñez‐Saucedo, B. E. Reyes‐Torres, K. Kortright‐Maldonado, E. K. Tenorio‐Aguirre, P. Rodríguez‐Henríquez, and F. D. Martínez‐Sánchez, “Acute Pancreatitis Associated With Systemic Lupus Erythematosus in a Young Female: A Diagnostic and Therapeutic Challenge,” *Clinical Case Reports* 12, no. 11 (2024): e9621, https://doi.org/10.1002/ccr3.9621.

There is a typo in the first author's surname. It was originally spelled as “Ordones‐Saucedo” but should be corrected to “Ordonez‐Saucedo.”

We apologize for this error.

